# Supplementary material for: The causal relationship between the human gut microbiota and pyogenic arthritis: a Mendelian randomization study
Source: Front Cell Infect Microbiol. 2024 Nov 26;14:1452480. doi: 10.3389/fcimb.2024.1452480 (PMC11629706; doi:10.3389/fcimb.2024.1452480)
Supplement: Supplementary file 1 [file DataSheet1.zip › mendelian test/ebi-a-GCST90027621.csv_forest.pdf]

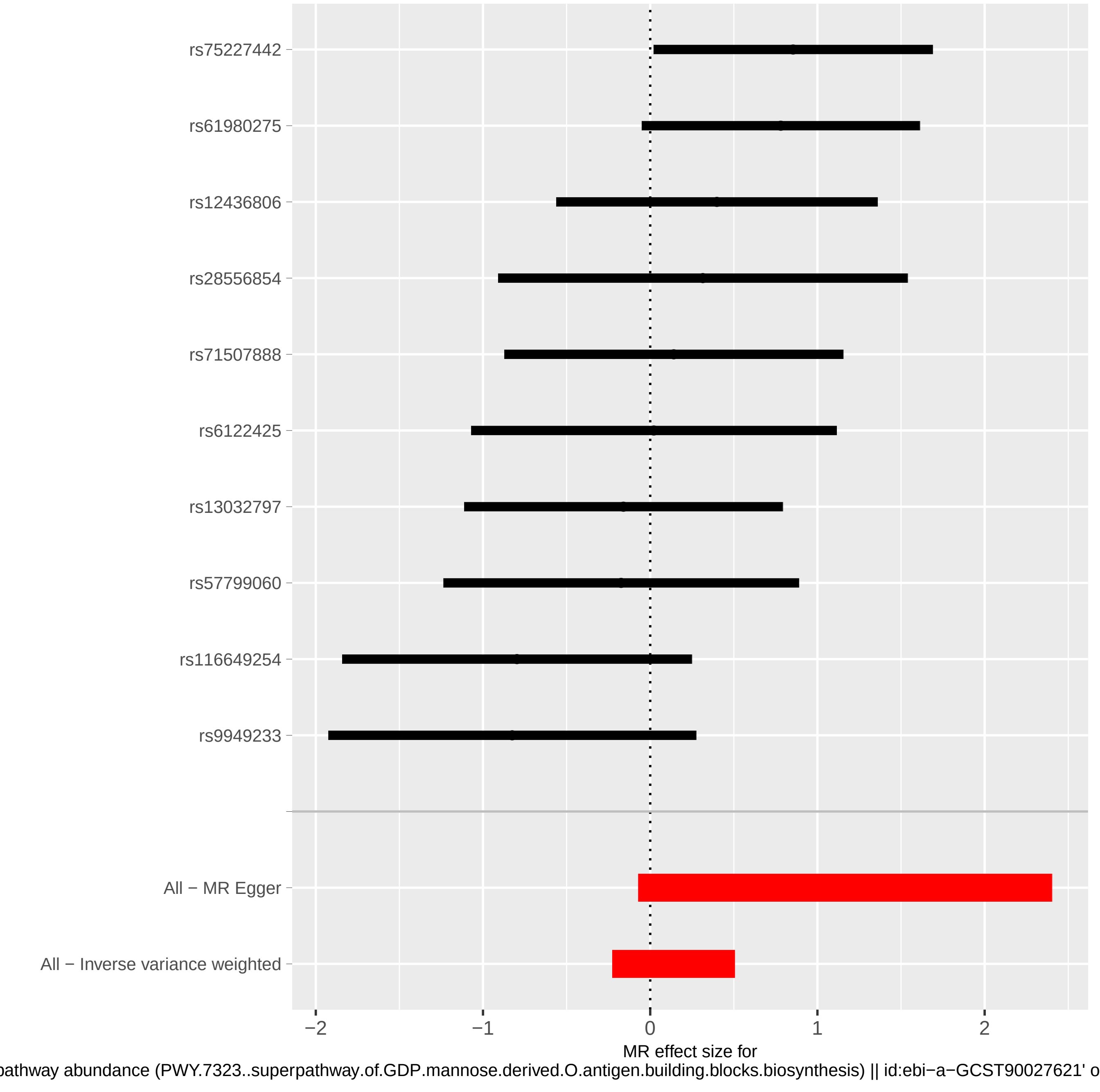
rs75227442
rs61980275
rs12436806
rs28556854
rs71507888
rs6122425
rs13032797
rs57799060
rs116649254
rs9949233
All − MR Egger
All − Inverse variance weighted
−2
−1
0
1
2
MR effect size for
pathway abundance (PWY.7323..superpathway.of.GDP.mannose.derived.O.antigen.building.blocks.biosynthesis) || id:ebi−a−GCST90027621' o
